# Combining multifaceted aspects of technology innovations through fuzzy clustering of multilayer networks

**DOI:** 10.1371/journal.pone.0334138

**Published:** 2025-10-14

**Authors:** Roy Cerqueti, Giovanna Ferraro, Raffaele Mattera, Saverio Storani

**Affiliations:** 1 Department of Social and Economic Sciences, Sapienza University of Rome, Rome, Italy; 2 GRANEM, SFR CONFLUENCES, University of Angers, Angers, France; 3 Department of Enterprise Engineering, University of Rome Tor Vergata, Rome, Italy; 4 Department of Mathematics and Physics, Luigi Vanvitelli University of Campania, Napoli, Italy; 5 Department of Legal and Economic Sciences, University of Rome Unitelma Sapienza, Rome, Italy; University of Foggia, ITALY

## Abstract

This study advances a novel multilayer network model to explore the connection between different aspects of Technological Innovation in European Union (EU) countries. We follow a fuzzy clustering approach and consider three variables: Research and Development (R&D), High-Tech Exports (HTE), and Human Resources in Science and Technology (HRST). We consider Eurostat data from 2018 to 2023. The variables form the layers, the EU countries are the nodes of the layers, and the weighted intra-layer links are assumed to increase with respect to the similarity of the countries in terms of the related variable. Interlayer connections are modeled probabilistically using a fuzzy clustering approach: two countries in different layers are strongly connected if they belong more probably to the same cluster in the related layers. The analysis offers insights into the patterns of EU countries in terms of Technological Innovation (TI) processes. The proposed framework allows its applicability to a wide set of real-world contexts.

## 1 Introduction

In recent years, Technological Innovation (TI) has emerged as a critical driver of future economic growth, due to its profound economic, social, and environmental implications (see [1–4]). The continuous evolution of technology is reshaping global economies and driving progress across all sectors [5,6]. These technological changes, particularly those that foster innovation and efficiency, are essential for the transition to a more knowledge-driven and sustainable global economy [7,8]. As economies increasingly rely on TI, analyzing and tracking the technological integration across European countries becomes essential for shaping long-term development strategies. This growing importance has made TI a central concern in policy and decision-making processes [9,10].

This paper focuses on the intersections of three critical components of TI: Research and Development (R&D) expenditure, High-Tech Exports (HTE), and Human Resources in the Science and Technology (HRST).

R&D plays a fundamental role in driving innovation, enabling the development of new technologies and scientific advancements with significant impacts across multiple sectors. It plays a critical role in addressing global challenges, from economic development to climate change, by generating solutions that enhance efficiency, sustainability, and competitiveness [11,12].

In parallel, HTE, particularly in biotechnology, digital technologies, and green innovation, is a pivotal factor to drive economic growth by creating jobs and promoting sustainable innovation [13]. These sectors facilitate the commercialization of research results and accelerate the scaling of innovative solutions [14].

Finally, the role of HRST is important, which underpins the effective deployment and advancement of both R&D and HTE. A highly skilled workforce in science and technology is crucial to translate research outcomes into practical applications, thereby driving productivity and innovation across industries [15,16].

R&D, HTE, and HRST represent aspects that are deeply connected to the socio-economic growth of countries. The literature provides evidence that countries investing more heavily in R&D typically experience higher levels of TI, leading to higher economic performance [17]. Similarly, the development of HRST is essential to support and amplify these effects [18,19].

In this study, we analyze the interrelations among these three pillars at the country level, considering their evolution over time. We begin with the observation that countries exhibit different behaviors across the examined variables, and such heterogeneity is not only cross-sectional but also temporal. Our empirical investigation considers the important instance of the European Union (EU) countries, drawing on annual data from Eurostat for the period 2018 to 2023, a timeframe that includes the disruptive effects of the COVID-19 pandemic. The selected variables, R&D, HTE, and HRST, are those officially designated by Eurostat. Their definitions are summarized in Table 1.

**Table 1:** Notations and description of the considered variables.

Notation	Variables	Description
R&D	R&D total expenditure	Measures total expenditure on Research & Development (R&D) as a percentage of GDP. R&D includes creative work undertaken systematically to increase the stock of knowledge in science, culture, and society, and its use to develop new applications.
HTE	High tech exports	Measures the percentage of a country’s total exports that consists of high-technology products. This indicator reflects the degree of specialization in technologically advanced products, showing how much of a country’s export activity is oriented toward high-tech goods.
HRST	Human resources in science and technology	Measures the percentage of the active population aged 25–64 that is classified as HRST, i.e., those who have successfully completed tertiary education or are employed in science and technology occupations. This indicator reflects the availability of highly skilled human capital in a country.

Although it may seem intuitive that countries with advanced TI exhibit similar values of the dimensions considered, the reality is more nuanced. The outcomes of TI are influenced by a variety of contextual factors, including economic structures, educational systems, and policy frameworks, which differ between countries and evolve over time [20,21].

Observing the evolution of R&D, HTE, and HRST enables the identification of countries that pursue similar technological strategies and offers insight into how these approaches influence the trajectories of economic and social development [22,23].

The intersections between these dimensions are complex and multifaceted, reflecting broader trends in the convergence of knowledge-intensive industries, services, and technologies. To foster innovation, accelerate digital transformation, and maintain competitiveness, it is important to ensure that R&D efforts, particularly in the industrial sector, are closely integrated with the High-Tech Industry and Knowledge-Intensive Service exports sectors, which are fundamental for defining the HTE variable [24]. Strategic cooperation, knowledge exchange, and skill development between sectors are crucial to navigate an increasingly complex and technology-driven global economy.

R&D in the industrial sector is particularly oriented toward the development of new products, processes, and technologies to improve productivity, sustainability, and performance. However, realizing these innovations requires not only advanced knowledge and skilled labour, but also cross-sectoral collaboration.

High-tech manufacturing and knowledge-intensive services play complementary roles, supporting R&D by providing specialized capabilities. Firms in these sectors, such as management consultants, financial analysts, legal advisors, and data scientists, frequently collaborate with industrial entities to translate research into practical business strategies [25]. Moreover, the recruitment, training, and retention of qualified personnel are critical for advancing these innovations. Workers in technological sectors must have the necessary competencies in emerging tools such as Artificial Intelligence (AI), the Internet of Things (IoT), and automation technologies.

This paper aims to explore this heterogeneous and intricate landscape.

To investigate the relationships among R&D, HTE, and HRST, we adopt a multilayer network approach, which offers a robust framework for capturing complex interdependencies between variables. This methodology is particularly effective in identifying heterogeneous relationships across interconnected systems [26,27]. Due to their flexibility, multilayer networks have gained prominence in the analysis of social phenomena, enabling the representation of multiple types of dependencies within a unified structure [28].

They have been employed in diverse fields such as social interaction and group dynamics [29,30], disease modeling [31], financial systems [32,33], global waste trade [34], and social-environmental policy integration in the European Union countries [35].

Multilayer networks are also effective in modeling relationships within scientific communities, where layers may represent dimensions such as co-authorship or institutional affiliation [36]. By capturing inter-layer interactions, these networks uncover dependencies that single-layer models cannot, providing a deeper understanding of complex structures.

In this research, each layer represents one of the three variables (R&D, HTE, HRST), with nodes corresponding to EU countries. For each year from 2018 to 2023, we construct a multilayer network in which intra-layer link weights indicate the degree of similarity between countries based on the respective variable—the higher the similarity, the higher the corresponding weight. Inter-layer connections are established through a community detection process, linking countries that belong to the same cluster across different layers.

To detect communities in each layer we could perform several methods [37]. Among them, we can cite hierarchical [38], K-means [39,40], Voronoi tessellation [41], and fuzzy [42] clustering (for comparison among cluster algorithms, see [43]). In this paper, we detect communities in the layers by adopting the fuzzy approach in [42]. The motivation for implementing fuzzy clustering lies in the versatility of this procedure. Indeed, fuzziness allows for overlapping clusters and probabilistic memberships, hence leading to a more meaningful set of results than the mere crisp assignments (see, e.g. [41,44]). For example, studies like [45] and [46] exploit model-based fuzzy clustering with time series-specific distance metrics, while [47] incorporate spline coefficients for portfolio allocation models. More recently, [48,49] introduced advanced Fuzzy Cluster Analysis (FCA) methods using conditional moments, optimizing clustering precision.

In this paper, inter-layer links are created by assigning a weight coming from the member degree associated with clusters by considering that two nodes belong to the same cluster when they are taken in different layers. Therefore, the presence of an inter-layer link between two countries measures the evidence, derived from a fuzzy approach, that the countries belong to the same class for both variables. A high value of the weight of an inter-layer link suggests that the related countries adopt similar strategies in addressing the aspect represented by the respective variables.

The results of our analysis show deviations and similarities among EU countries in their technological development pathways over time.

This study contributes to the literature in several ways. First, it is among the few studies to use a multilayer network model to investigate the interplay between R&D, HTE, and HRST as different aspects of TI. Second, by incorporating both intra- and inter-layer analyses, it offers a detailed view of how these elements interact and affect each other. Finally, it provides insights into how technological capabilities translate into socio-economic outcomes across countries.

The rest of the paper is organized as follows: Sect 2 describes the dataset, time frame, and selected variable. Sect 3 outlines the methodology, detailing the multilayer network construction and community detection process. Sect 4 presents the empirical results and discussion, including methodological implications. Sect 5 concludes by summarizing the key findings and discussing the limitations of the proposed framework.

## 2 Data

A description of the variables under consideration and the notation used to deal with them can be found in Table 1. All the variables are expressed as percentages: R&D as a share of GDP, HTE as a share of total exports, and HRST as a share of the active population aged 25–64; for details on the notation, units of measurement, and descriptions, see Table 1. We investigated a panel data set from Eurostat, the Official Statistical Office of the European Union (further details are available at the Eurostat website: https://ec.europa.eu/eurostat). Although the spatial dimension is related to *N* (i=1,…,N) countries, the temporal dimension is discrete and captures *T* years (t=1,…,T). For all factors taken into account, the data has been properly handled to have a common inquiry period and a common set of countries.

As a result, the investigation term spans *T* = 6 years, from 2018 to 2023, and there are *N* = 27 countries that are being examined. We list them in Table 2 with their acronyms. The analyzed countries are all members of the European Union.

**Table 2:** Acronyms and corresponding EU countries.

Acronym	Country	Acronym	Country
AT	Austria	LT	Lithuania
BE	Belgium	LU	Luxembourg
BG	Bulgaria	LV	Latvia
CY	Cyprus	MT	Malta
CZ	Czechia	NL	Netherlands
DE	Germany	PL	Poland
DK	Denmark	PT	Portugal
EE	Estonia	RO	Romania
EL	Greece	SI	Slovenia
ES	Spain	SE	Sweden
FI	Finland	SK	Slovakia
FR	France	HR	Croatia
HU	Hungary	IE	Ireland
IT	Italy		

Table 3 reports the Pearson correlation matrix for R&D, HTE, and HRST across the period 2018–2023.

**Table 3:** Pearson correlation matrix showing the relationship between RD, HTE, and HRST across the period 2018–2023.

	R&D	HTE	HRST
R&D	1.0000	0.6392	0.8875
HTE	0.6392	1.0000	0.6520
HRST	0.8875	0.6520	1.0000

Table 4 presents descriptive statistics for the considered variables (R&D, HTE, and HRST) from 2018 to 2023.

**Table 4:** Descriptive statistics for selected variables from 2018 to 2023.

Variables	Statistics	2018	2019	2020	2021	2022	2023
**R&D**	Min.	0.50	0.47	0.46	0.47	0.46	0.52
	1st Qu.	0.94	1.04	1.11	1.06	1.05	1.05
	Median	1.35	1.39	1.50	1.43	1.44	1.49
	Mean	1.61	1.67	1.75	1.74	1.74	1.76
	3rd Qu.	2.15	2.17	2.27	2.22	2.20	2.16
	Max.	3.35	3.40	3.50	3.42	3.47	3.57
**HTE**	Min.	4.30	3.98	4.60	4.94	4.74	4.97
	1st Qu.	7.26	7.395	7.21	7.20	7.105	6.775
	Median	9.76	9.70	9.82	9.91	9.02	8.89
	Mean	11.70	11.72	12.21	12.88	12.238	12.06
	3rd Qu.	14.94	14.53	15.16	15.10	15.01	14.64
	Max.	34.49	34.87	38.57	42.49	43.50	42.46
**HRST**	Min.	27.90	28.20	28.40	30.40	31.40	29.90
	1st Qu.	39.55	39.55	40.50	41.95	42.40	42.55
	Median	47.40	48.20	50.60	52.00	51.80	51.00
	Mean	47.07	48.03	49.20	50.36	50.88	51.09
	3rd Qu.	53.30	54.05	55.90	56.75	56.80	58.10
	Max.	61.20	63.70	65.00	68.10	69.20	68.10

For R&D, there is an oscillating behavior of the mean with a slight increase from 1.61 in 2018 to 1.76 in 2023, indicating a general growth in research and development investments across European countries. The maximum values also increased from 2018 to 2023, suggesting that some countries have significantly intensified their efforts in this area.

Regarding HTE, the average values exhibit an inverse U-shaped behavior, increasing from 11.70 in 2018 to 12.06 in 2023 but with a maximum average value in 2021. The maximum values show great variability but with a substantially increasing trend (rising from 34.49 to 42.46). This reflects a substantial increase in high-tech exports for leading countries. However, the first quartile shows a decline over time, suggesting that exports are unevenly distributed and that some countries have fallen behind.

Finally, for HRST, the mean displays a steady rise during the period, increasing from 47.07 in 2018 to 51.09 in 2023. Also the maximum is substantially increasing, moving from 61.20 in 2018 to 68.10 in 2023. These trends highlight an improvement in human resources employed in science and technology, reflecting a growing focus on the training and employment of highly skilled personnel.

Overall, the data suggest a trend toward greater technological integration, although significant disparities persist among countries, as indicated by the uneven distribution of extreme values.

Finally, Table 3 shows the correlation matrix for our three variables. R&D and HRST exhibit a strong positive correlation (0.8875), indicating that countries with higher R&D expenditure generally have a larger share of highly skilled human resources in science and technology. R&D and HTE, as well as HTE and HRST, show moderate positive correlations (0.6392 and 0.6520, respectively), suggesting that while investment and human capital are related to high-tech exports, other factors also play an important role in determining a country’s export performance.

## 3 Methodology

This section discusses the network model adopted for carrying out the connectedness analysis and the methodological procedure used for clustering network nodes (i.e., EU countries) and creating the inter-layer connections.

### 3.1 The multilayer network model

We assume that the *K* = 3 variables R&D, HTE, and HRST are the instruments used for building three distinct layers in a multilayer network structure, all sharing the same set of nodes, which are the *N* = 27 EU countries. These countries are collected in a set V={1,…,i,…,27}. A description of the variables is provided in Table 1. For each time *t*, we construct a multilayer network comprising 27 nodes. For simplicity, we describe the network at a generic time *t* and omit the time index from the notation.

The multilayer network is represented as follows:

𝒩=(𝒢,𝒞), (1)

where $𝒢=\{G_{1},G_{2},G_{3}\}$ denotes the set of three distinct graphs, each corresponding to one of the variables from Table 1. For each k=1,2,3, the *k*-th graph $G_{k}=(V,W_{k})$ is characterized by a weighted adjacency matrix $W_{k}=(w_{k}(i,j)|i,j\in V)$, representing the connections between nodes (countries) in the *k*-th layer of the network.

The set 𝒞 includes the weighted links between countries across different layers, defined as $𝒞=E_{hk}(i,j)\in [0,1]|h,k=1,2,3;\;h\neq k\text{ and }i,j\in V$. For additional information see, [50], and [51].

For each layer *k*, we construct the weighted adjacency matrix *W*_*k*_ based on a similarity measure. Specifically, the strength of the connection between nodes *i* and *j* is inversely proportional to the distance between their corresponding values for the *k*-th variable. In other words, the more similar two nodes are in terms of the *k*-th variable, the stronger the connection between them in the *k*-th layer of the multilayer network defined by Eq (1).

Let *x*_*k*_(*i*) and *x*_*k*_(*j*) represent the values of the *k*-th variable for countries *i* and *j*, respectively. The normalized distance $d_{k}:V^{2}\to [0,1]$ between these nodes is defined as follows:

$$d_{k}(i,j)=\frac{\left|x_{k}(i)-x_{k}(j)\right|}{\max|x_{k}(u)-x_{k}(v)|:u,v\in V}.$$ (2)

Then, the weight *w*_*k*_(*i*,*j*) is defined as:

$$w_{k}(i,j)=\frac{2}{d_{k}(i,j)+1}-1,$$ (3)

which ensures that *w*_*k*_(*i*,*j*) decreases as the distance *d*_*k*_(*i*,*j*) increases. Specifically, the greater the distance between nodes *i* and *j*, the weaker the connection between them. In the extreme cases, when *d*_*k*_(*i*,*j*) = 0 or *d*_*k*_(*i*,*j*) = 1, the corresponding weights are *w*_*k*_(*i*,*j*) = 1 and *w*_*k*_(*i*,*j*) = 0, respectively.

### 3.2 Centrality measures for intra-layer analysis

To analyze the patterns and relationships within each layer of the multilayer network model, we exploit three different centrality measures: strength, closeness, and clustering coefficient. These measures provide insights into the structural characteristics and roles of individual countries within the various layers of the network.

The strength of a node *i* in a layer *k* represents the weighted sum of the connections between the node and all the other nodes in the layer. For a given node *i*, the strength of *i* is defined as:

$$s_{k}(i)=\sum_{j\in V,j\neq i}w_{k}(i,j),$$ (4)

where *w*_*k*_(*i*,*j*) is as in (3). The strength quantifies the overall intensity of a node’s connections within a layer, identifying the most “central" nodes in terms of their connectivity. This concept, which reflects the extent of a node’s involvement in the network, is widely recognized in the literature as a fundamental indicator of node centrality [52–54].

The closeness of a node *i* in a layer *k* is the inverse of the strength, and it indicates how easily a node can be linked to the other nodes in the network. The closeness of *i* is:

$$c_{k}(i)=\frac{1}{\sum_{j\in V,j\neq i}w_{k}(i,j)}.$$ (5)

The formula (5) implies that higher weights correspond to shorter closeness, and vice versa. Consequently, closeness centrality in our framework reflects the relevance of the nodes in terms of the connections with their adjacent nodes (see e.g., [55,56]).

The clustering coefficient of a node *i* in layer *k* measures the tendency of *i* to form tightly-knit groups with its neighbors, reflecting the local cohesion of the network. We adopt the clustering coefficient defined by [57], which is given by:

$$cc_{k}(i)=\frac{1}{d_{i}^{\left(k\right)}(d_{i}^{\left(k\right)}-1)}\sum_{j\neq i}\sum_{k\neq i,j}{\left(w_{k}(i,j)w_{k}(i,h)w_{k}(j,h)\right)}^{1/3},$$ (6)

where $d_{i}^{\left(k\right)}$ is the degree of node *i* in layer *k*, i.e. the number of neighbors of node *i* in layer *k*. The clustering coefficient *cc*_*k*_(*i*) quantifies the local density of connections among the neighbors of node *i*, where a higher value indicates a higher tendency for node *i* to form cohesive clusters with its neighbors.

To obtain a global measure of the centrality and cohesion within each layer, we calculate the average of the individual node-level measures. This provides a more comprehensive summary of the overall network structure. Specifically, for each measure, the global value is computed as the average across all nodes in the network.

Specifically, the global strength $\langle s_{k}\rangle$ is the average strength of all nodes in layer *k*. It is calculated as:

$$\langle s_{k}\rangle =\frac{1}{N}\sum_{i\in V}s_{k}(i).$$ (7)

Similarly, the global closeness $\langle c_{k}\rangle$ is the average closeness of all nodes in layer *k*. It is given by:

$$\langle c_{k}\rangle =\frac{1}{N}\sum_{i\in V}c_{k}(i).$$ (8)

Finally, the global clustering coefficient $\langle cc_{k}\rangle$ is the average of the local clustering coefficients across all nodes in the network, as follows:

$$\langle cc_{k}\rangle =\frac{1}{N}\sum_{i\in V}cc_{k}(i).$$ (9)

By calculating these global averages, we obtain a unified measure of the network’s overall connectivity (strength), accessibility (closeness), and local cohesion (clustering coefficient). These metrics offer insights into the overall behavior of the network and allow for comparative analysis across different layers.

### 3.3 Community detection procedure and inter-layer link formation

The employed community detection procedure is based on the similarity of the nodes in terms of the weights in (3). We include the presence of uncertainty in the assignments of countries in clusters by selecting a fuzzy clustering approach, through a Fuzzy C-Means (FCM) algorithm (for an introduction of the procedure and the generalization see respectively, [58,59]).

We identify four sources of advantages in fuzzy clustering. First, the trajectories of EU countries across R&D, HTE, and HRST are not clear-cut. A country may be advanced in one of these variables but only partially developed in the other dimensions. Therefore, exclusive membership to a single cluster would not capture such nuanced profiles. Second, hard clustering forces each country into a single class, leading to a rigid partition and potential information loss in heterogeneous contexts such as the EU. This is a remarkable limitation of crisp clustering when compared to fuzzy procedures. Third, fuzzy clustering admits the overlapping of cluster memberships, assigning degrees of belonging to multiple groups. This provides a more realistic representation of the hybrid innovation strategies of the countries and avoids rigidly misleading classifications. Fourth, from a methodological perspective, fuzzy clustering yields membership degrees that we directly exploit to build weighted inter-layer connections. Such interdependencies—which are particularly reasonable in our context—could not be derived from a hard clustering approach. Moreover, the fuzziness parameter enables us to control the degree of uncertainty, enhancing robustness and flexibility.

In the considered fuzzy clustering context, the objective function to be minimized is:

$$J_{v}=\sum_{i=1}^{N}\sum_{m=1}^{M}u_{mi}^{v},\|x_{i}-c_{m}{\|}^{2},$$ (10)

where *M* is an integer representing the number of clusters, ‖a−b‖ is the Euclidean distance between vectors *a* and *b*, $x_{i}\in \mathbb{R}$ is the value of the considered attribute of node *i*, *c*_*m*_ represents the centroid of the *m*-th cluster, $u_{mi}\in [0,1]$ is the membership degree of node *i* when taken into cluster *m*, and *v* > 1 is a parameter associated with the fuzziness. The role of *v* is to describe the degree of overlap of the clusters; such a degree is high when *v* is large. Moreover, by definition, one has the constraint


$$\sum_{m=1}^{M}u_{mi}=1,\;\forall i\in V.$$

The control variables for the minimization are the centroids and the membership degrees.

The optimization problem leads to the update of the *m*-th centroid according to this formula

$$c_{m}=\frac{\sum_{i=1}^{N}u_{mi}^{v}x_{i}}{\sum_{i=1}^{N}u_{mi}^{v}},$$ (11)

and membership degree *u*_*mi*_ given by

$$u_{mi}=\frac{1}{\sum_{k=1}^{M}{\left(\frac{\left\|x_{i}-c_{m}\right\|}{\left\|x_{i}-c_{k}\right\|}\right)}^{\frac{2}{v-1}}}.$$ (12)

The sequence of updates of centroids and membership degrees proceeds until the convergence of the algorithm.

The selection of *M* is performed by using the Fuzzy Silhouette (FS) criterion (for the methodological details, see [60]). In doing so, we are in line with authoritative contributions in the literature (see e.g., [61,62]).

### 3.4 Inter-layer links

To model inter-layer links, we use the results of fuzzy clustering to define the probability of a connection between nodes in different layers. We specify the layer *k* in the membership degree of node *i* in cluster *m* with the notation *u*_*mi*_(*k*). We assume that *M*_*k*_ is the number of clusters in layer *k*. We consider the distance between country *i* and *j* in layer *k* as

$$d_{k}(i,j)=\frac{1}{2}\sum_{m=1}^{M_{k}}{\left(u_{mi}(k)-u_{mj}(k)\right)}^{2}.$$ (13)

The inter-layer link between nodes *i* and *j* in layers *h* and *k* (with h≠k) is assumed to be based on the following inter-layer perturbed distance:

$${\tilde{d}}_{hk}(i,j)=\alpha |d_{k}(i,j)-d_{h}(i,j)|+(1-\alpha )\max\{d_{k}(i,j),d_{h}(i,j)\},$$ (14)

with α∈[0,1].

By definition, ${\tilde{d}}_{hk}(i,j)={\tilde{d}}_{kh}(i,j)$. This means that the distance between *i* and *j* when placed on different layers is symmetric, with no relevance to the specific layer where each node is placed. Moreover, ${\tilde{d}}_{hk}(i,j)\in [0,1]$, for each i,j∈V and h,k=1,2,3,h≠k.

The rationale of the formula (15) is as follows: the distance between two nodes *i* and *j* in layers *k* and *h* is due to the aggregation of the absolute difference between *d*_*k*_(*i*,*j*) and *d*_*h*_(*i*,*j*) and a perturbation term associated to the highest value between them. The parameter *α* is a quantity that provides the adjustment between these two components, more focused on the first (case *α* close to 1) or the second (case *α* close to 0). In general, ${\tilde{d}}_{hk}(i,j)$ emphasizes similar distances between *i* and *j* in the considered layers (first component) by simultaneously penalizing the dissimilarity in at least one of the layers (second component). Substantially, once *α* is fixed, we can say that ${\tilde{d}}_{hk}(i,j)$ is close to zero when $d_{k}(i,j)\sim d_{h}(i,j)\sim 0$. In this case, it is interesting to observe that *i* and *j* have similar behaviors in terms of the variables defining the layers. If $d_{k}(i,j)\sim d_{h}(i,j)\sim 1$—i.e., the gap between *i* and *j* is the same in the considered layers and it is of high value—then ${\tilde{d}}_{hk}(i,j)$ is close to 1−α. In the case of $d_{k}(i,j)\sim 1$ and $d_{h}(i,j)\sim 0$, then ${\tilde{d}}_{hk}(i,j)$ is close to 1. In the corner case α=0, the distance ${\tilde{d}}_{hk}(i,j)$ depends solely on the maximum between *d*_*k*_(*i*,*j*) and *d*_*h*_(*i*,*j*). In this case, similar values of *d*_*k*_(*i*,*j*) and *d*_*h*_(*i*,*j*) do not necessarily lead to a small value of ${\tilde{d}}_{hk}(i,j)$. What is relevant is the value of *d*_*k*_(*i*,*j*) and *d*_*h*_(*i*,*j*). Therefore, the case α=0 penalizes the intra-layer distance by assigning a large value of ${\tilde{d}}_{hk}(i,j)$ to nodes *i* and *j* having a high distance at least in one of the layers *h* or *k*.

When α=1, the distance ${\tilde{d}}_{hk}(i,j)$ depends solely on the absolute difference $\left|d_{k}(i,j)\;-\;d_{h}(i,j)\right|$. In this case, similar values of *d*_*k*_(*i*,*j*) and *d*_*h*_(*i*,*j*) lead to ${\tilde{d}}_{hk}(i,j)$ close to zero, without any relevance in their values. Differently, a high dissimilarity between *d*_*k*_(*i*,*j*) and *d*_*h*_(*i*,*j*) gives a high value of ${\tilde{d}}_{hk}(i,j)$.

The weight between *i* and *j* in layers *k* and *h* is then

$$E_{hk}(i,j)=\frac{2}{{\tilde{d}}_{hk}(i,j)+1}-1.$$ (15)

By construction, $E_{hk}(i,j)\in [0,1]$; furthermore, it decreases with respect to ${\tilde{d}}_{hk}(i,j)$, with *E*_*hk*_(*i*,*j*) = 0 when ${\tilde{d}}_{hk}(i,j)=1$ and *E*_*hk*_(*i*,*j*) = 1 when ${\tilde{d}}_{hk}(i,j)=0$.

## 4 Results and discussion

Table 5 provides an overview of global network metrics (strength, clustering coefficient, and closeness) for the variables R&D, HTE, and HRST across the years 2018 to 2023, offering insights into the evolution of intra-layer connections over time. These metrics are computed following the criteria detailed in the Sect 3.2.

**Table 5:** Descriptive statistics of global network metrics (strength, clustering coefficient, and closeness) for each layer *k* from 2018 to 2023, for the variables R&D, HTE, and HRST. The table presents the evolution of these metrics over time, offering insights into the global structure and time-varying network of each layer.

Variables	Metrics	2018	2019	2020	2021	2022	2023
R&D	Strength	13.820	13.909	14.030	13.781	14.071	14.077
	Clust. Coeff.	0.487	0.485	0.494	0.481	0.496	0.495
	Closeness	0.167	0.156	0.145	0.156	0.143	0.148
HTE	Strength	17.157	17.180	17.460	17.562	18.197	17.950
	Clust. Coeff.	0.635	0.634	0.645	0.647	0.673	0.660
	Closeness	0.168	0.165	0.177	0.207	0.206	0.251
HRST	Strength	14.865	15.119	15.210	15.249	15.098	15.239
	Clust. Coeff.	0.538	0.549	0.553	0.559	0.550	0.555
	Closeness	0.115	0.108	0.107	0.102	0.104	0.104

The global strength remains relatively stable for the R&D and HRST layers. For R&D, the values oscillate around 14, indicating consistent connectivity, while HRST shows minor fluctuations around 15, reflecting stable intra-layer interactions. Differently, the HTE layer shows a gradual increase in strength, peaking in 2022 before slightly declining in 2023, suggesting an overall strengthening of relationships within this layer over time.

The global clustering coefficient reveals distinct trends. The R&D layer shows minor variations, with values consistently close to 0.5, reflecting steady local clustering. For the HTE layer, there is a steady increase, with values rising from 0.635 in 2018 to 0.673 in 2022, indicating an increasing tendency for nodes to form tight groups. The HRST layer also exhibits a slight upward trend, with values increasing from 0.538 in 2018 to 0.555 in 2023, suggesting slight improvements in local connectivity.

The global closeness presents a more varied picture. For the R&D layer, closeness decreases initially, from 0.167 in 2018 to 0.145 in 2020, before recovering slightly to 0.148 in 2023. The HTE layer shows a notable increase, with its value rising significantly from 0.177 in 2020 to 0.251 in 2023. Conversely, the HRST layer shows a steady decrease over time, reaching a 0.104 value in 2023. According to our global closeness definition detailed in Sect 3.2, these metrics depict better integration for variables R&D and HRST than HTE.

Overall, the HTE layer emerges as the most dynamic, with noticeable increases in all metrics. The increase in the global clustering coefficient indicates stronger local cohesion, with nodes forming more densely connected clusters. However, the increase in global closeness suggests reduced global accessibility, likely due to weaker connections between clusters or the loss of strategic hubs and links that previously ensured shorter paths. This reflects a network that is more fragmented globally but more cohesive locally. In comparison, the R&D and HRST layers demonstrate a more stable behavior, with only minor fluctuations observed over the analyzed period. Despite these subtle variations, the overall trend of the metrics suggests a clear trajectory toward increasing integration within these layers. This indicates that while the structural dynamics of these networks remain relatively consistent, they are gradually aligning more closely, reflecting a steady enhancement in their interconnectedness and a potential harmonization of the underlying systems they represent.

**Fig 1:**
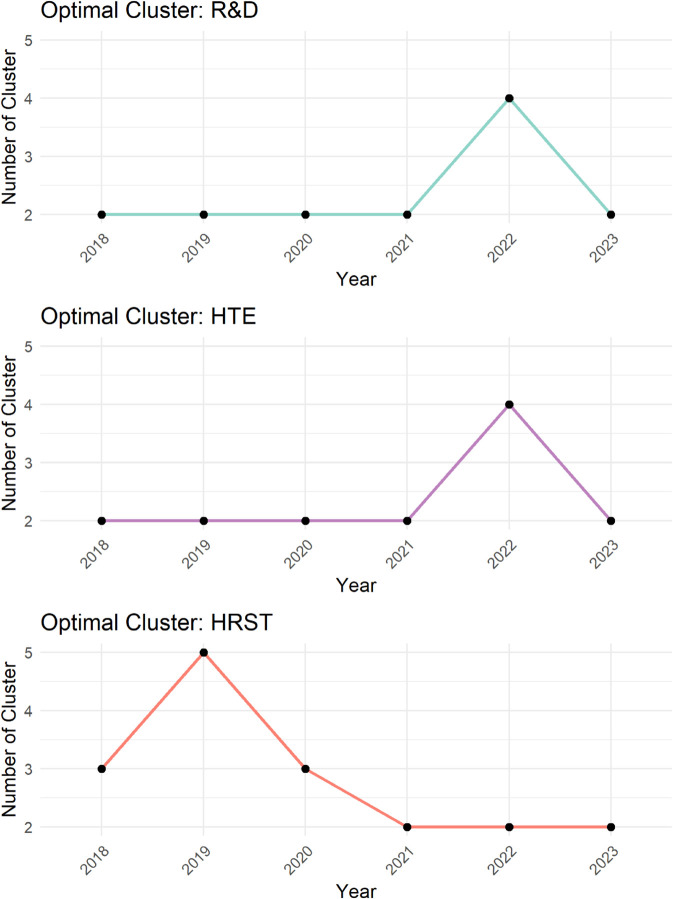
Fuzzy Silhouette values for each variable over time. Each panel shows the optimal number of clusters, ranging from 2 to 5, highlighting temporal variations and clustering dynamics for each variable.

The results shown in Fig 1 depict the optimal number of clusters over time according to the FS criterion detailed in Sect 3, which reveals distinct temporal patterns in the three variables the R&D, HTE, and HRST.

For R&D and HTE, the number of clusters remains stable at 2 from 2018 to 2021, reflecting consistent structural cohesion and well-defined groupings over this period. However, in 2022, a sudden increase to 4 clusters suggests a temporary diversification or fragmentation within the two layers, potentially driven by exogenous shocks or significant changes in the system’s dynamics. This shift might indicate the emergence of subgroups or increased differentiation in the network structure. By 2023, the number of clusters reverts to 2, implying a restoration of the earlier structural patterns and possibly reflecting a resolution of the transient changes observed in 2022.

In contrast, HRST displays greater variability in the number of clusters, starting with 3 in 2018, increasing to 5 in 2019, and then fluctuating between 3 in 2020 and 2 from 2021 onward. This pattern suggests a more dynamic network structure, with notable changes in clustering tendencies during the early years of the period analyzed. The initial increase in the number of clusters could indicate a phase of network diversification or the emergence of distinct substructures. The subsequent reduction to 2 clusters in 2021 through 2023 likely reflects a process of consolidation, where previously distinct clusters merged or became less distinct, leading to a more integrated and unified network structure.

These results underscore the dynamic nature of clustering across different network layers, characterized by alternating periods of stability and significant structural transitions. Such transitions may reflect systemic changes or external influences affecting the networks in diverse ways. The FS criterion, calculated based on distances between the 27 EU countries, provides robust validation for these patterns. These distances reflect the similarity among countries concerning specific variables, with a higher optimal number of clusters indicating greater heterogeneity. Notably, peaks in 2022 for R&D and HTE, and in 2019 for HRST, suggest events that may have driven increased heterogeneity during these periods. By 2023, however, a clear convergence emerges across all variables, highlighting a trend of growing integration and homogenization among EU countries within the context of the Union.

The second row in the aforementioned figures visualizes connections across three pairs of variables for the year 2023. This layer of analysis adds a crucial dimension to understanding the interplay between variables. In these visualizations, the color of the nodes continues to reflect the variable or layer they belong to, while the color gradient of the links captures the strength of inter-layer connections between countries, as described in Sect 3.4. These weights are calculated using Eq 15, emphasizing the underlying relationships between layers. This visualization not only highlights the connections but also uncovers the structural nuances of how countries interact across different variables. At the end, the third row in the figures, consistent with the inter-layer representation, presents heatmaps that provide a more compact and quantitative representation of the connection strengths between countries across two different layers or variables. In these heatmaps, the color intensity corresponds to the strength of the connections, with darker tones indicating stronger links. These visual representations allow for an intuitive comparison of connection patterns, making it easier to identify clusters of countries or variables with particularly strong interdependencies.

**Fig 2:**
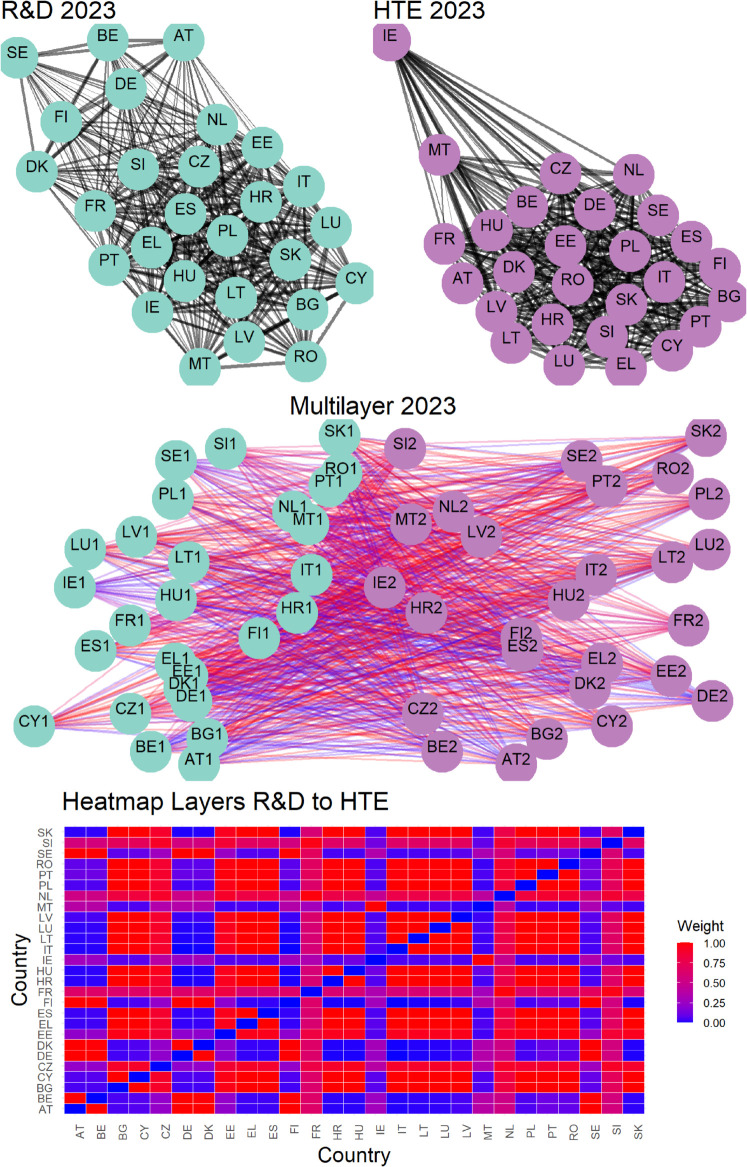
Visualization of multilayer network connections. The first row shows two networks corresponding to two variables, highlighting intra-layer connections. The second row illustrates the multilayer structure, showcasing inter-layer connections between nodes across networks. The third row presents a heatmap that captures inter-layer connections, with link intensity proportional to weight, as indicated by the color gradient.

**Fig 3:**
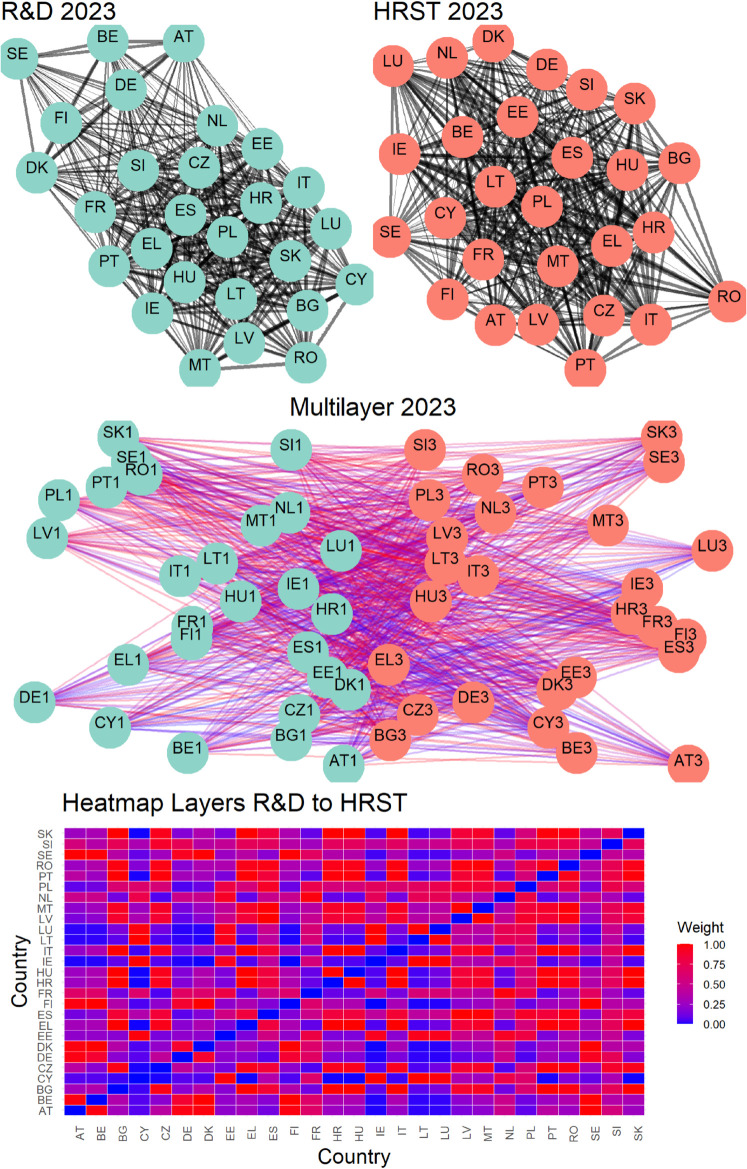
Multilayer network connection visualization. Intra-layer connections are highlighted by the top row, which displays two networks that correspond to two variables. The multilayer structure is depicted in the second row, which shows inter-layer interactions between nodes in various networks. With link intensity proportionate to weight, as seen by the color gradient, the third row displays a heatmap that shows inter-layer connections.

**Fig 4:**
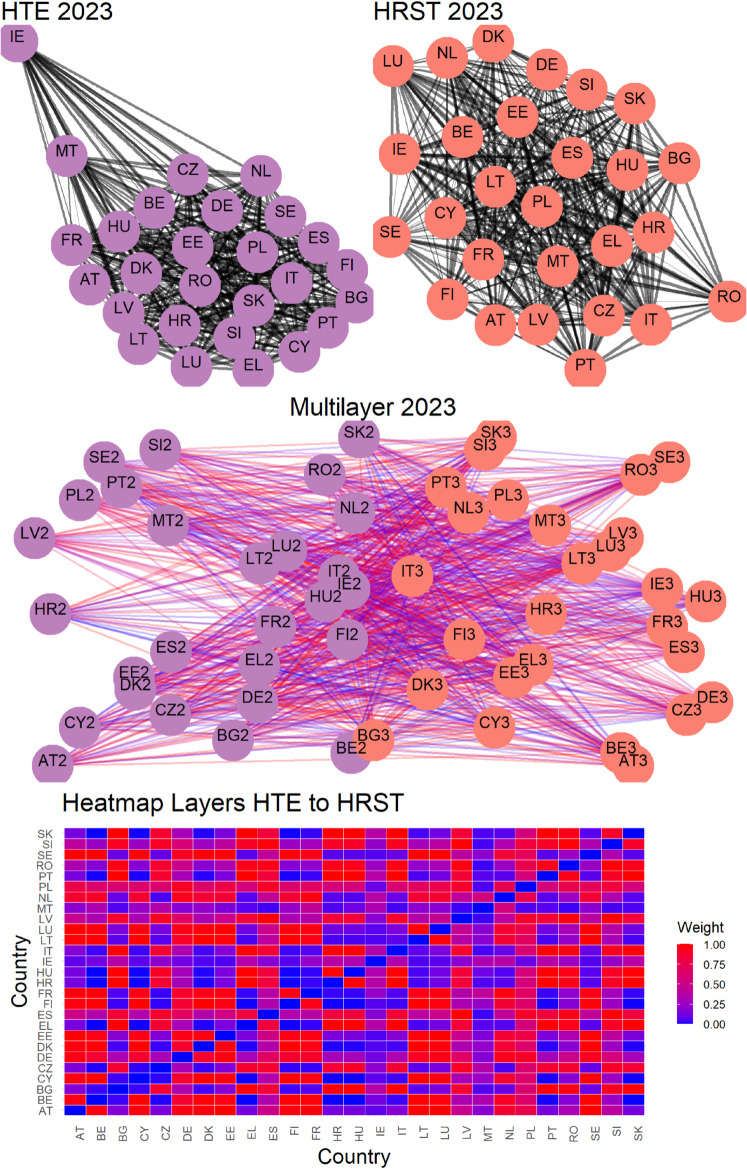
Visualization of intra-layer and inter-layer network connections. Detail are specified in Figs 2, and 3.

Overall, these figures offer a comprehensive view of the networks, clarifying both the intra-layer and inter-layer dynamics. The combination of visual and quantitative tools ensures a deeper understanding of the relationships at play, enhancing the interpretability of the data. Having said that, is necessary to specify that Figs 2, 3, and 4 refer only to the latest year in the available data so that we also provide a time-varying representation in the following.

**Fig 5:**
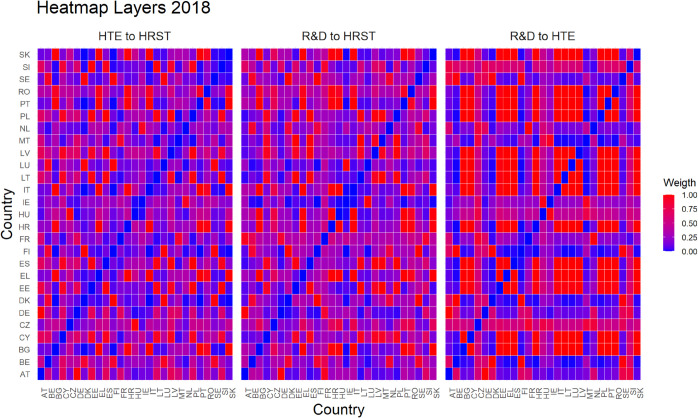
Heatmap for three different pairs of variables or layers in 2018. The columns and rows represent the 27 EU countries, while the color indicates the strength of the connection between countries across different layers, as defined by Eq 15.

**Fig 6:**
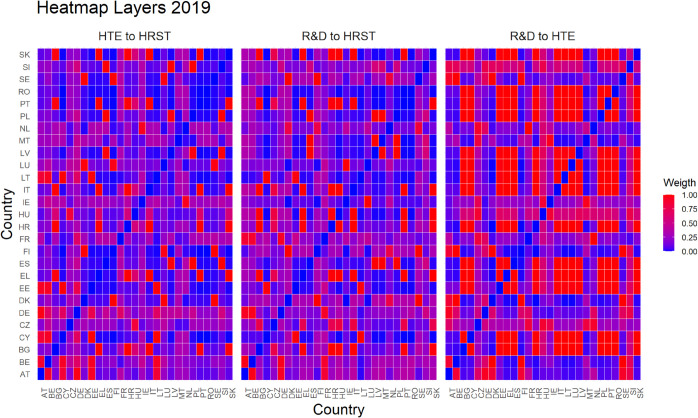
Heat map for three pairs of variables or layers in 2019. Rows and columns represent the 27 EU countries, with the gradient indicating connection strength between countries across layers.

**Fig 7:**
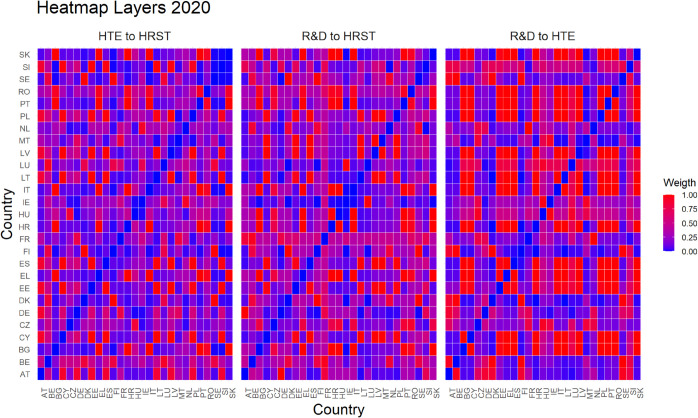
Heat map for three pairs of layers in 2020. The gradient represents the connection strength between the 27 EU countries, as detailed in Figs 5 and 6.

**Fig 8:**
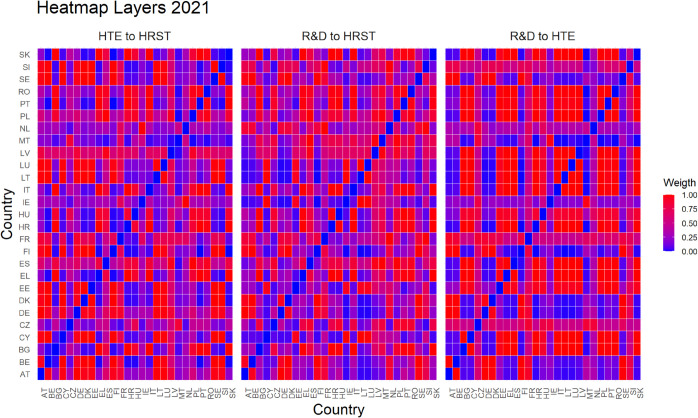
As displayed in Figs 5, 6, and 7, this heat map illustrates the connections between countries for three pairs of variables or layers in 2021. The gradient shows the strength of these links among the 27 EU countries.

**Fig 9:**
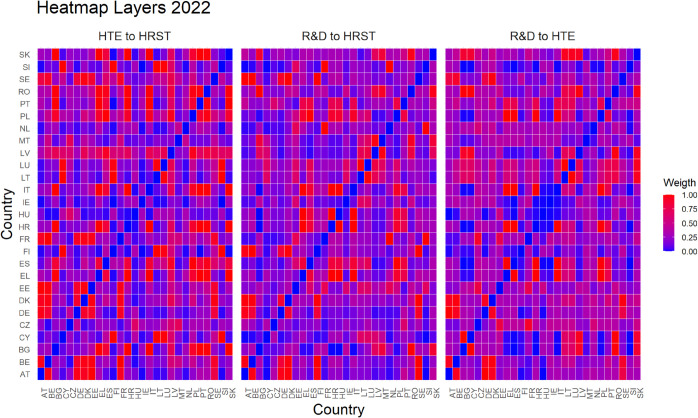
This heat map focuses on 2022, highlighting the connection intensities across three variable pairs among the 27 EU countries.

**Fig 10:**
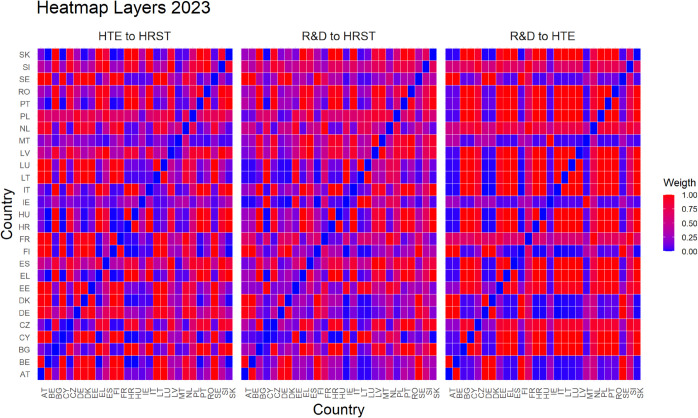
In 2023, as depicted in Figs 5, 6, 7, 8, and 9 the heat map captures the strength of relationships between the 27 EU countries across three different layers.

Finally, Figs 5–10 report all cases analyzed in this paper. In particular, we observe that in 2018 and 2019 the colors of the heatmaps relating to the pairs of layers are very similar (see Figs 5 and 6). This indicates that the link strength between countries in the considered layers remains substantially stable. However, in both years, for the R&D-HTE layer pairs, the heatmaps show a greater number of red cells. This suggests that the links between countries in the two layers are stronger than the other pairs of variables. Likewise, a similar behavior is observed in 2020 (see, Fig 7), where the heatmaps show a similar distribution of colors among the connections. Differently, in Fig 8, related to 2021, there is an intensification of the red cells compared to 2020, indicating an increase in inter-layer connections between countries for all layer pairs. As time progresses, in 2022, the heatmaps are characterized by blue cells, signaling a weakening of the intensity of the interlayer connections compared to the previous year (see Fig 9). Finally, in Fig 10, related to 2023, there is a noticeable increase in red cells, indicating stronger inter-layer connections. This behavior is consistent across all layer pairs. Additionally, there is a larger population of red cells compared to all previous years in every layer pair.

In light of the observations from the heatmaps, we can put forward conjectures about a possible relationship between the number of optimal clusters, observed in Fig 1, and the strength of the connections between the various layers. For example, we observe that the variables R&D and HTE are more strongly connected compared to other pairs of variables throughout the investigated period. In fact, for the variable pairs HRST-R&D, and HRST-HTE, we observe a weaker connection, except for the years 2021 and 2023 (see Figs 8 and 10). These specificities seem to be related to the number of clusters observed over time. A higher number of optimal clusters could indicate greater heterogeneity in the behavior of countries with respect to the variables, thereby affecting the strength of connections between layers.

Empirical experiments have been carried out by selecting α=0.5, which represents a balance parameter treating fairly the two components of the distance ${\tilde{d}}_{hk}$. This choice is grounded on the absence of prior knowledge about the investigated phenomena—even if our approach is rather general and allows other settings. Interestingly, the corner cases α=0 and α=1 belong to the range of possible opportunities. They can be efficiently used when one of the terms of the inter-layer perturbed distance does not play any role in the specific context under investigation. This is not the case for us. Looking at heatmaps in Figs 5–10, the red color means a strong connection between countries *i* and *j* in layers *h* and *k*. When taking α=0.5, then the red color represents a fair combination of both terms in (14). This means that we can identify the pairs of nodes that exhibit similar behavior between the two layers considered and, at the same time, have a small distance in both layers.

## 5 Conclusive remarks

In this study, we develop a novel multilayer network model to explore the relationships between different aspects of Technological Innovation (TI) across European Union (EU) countries and for the period 2018-2023. The model incorporates three key variables—Research and Development (R&D), High-Tech Exports (HTE), and Human Resources in Science and Technology (HRST)—which serve as the layers of the multilayer network. Countries are represented as nodes, with weighted intra-layer connections indicating the degree of similarity between countries within each variable. Stronger connections reflect greater similarity among countries in the same layer.

For inter-layer connections, we apply a fuzzy clustering approach, which allows us to build weighted links between countries across different layers. This method assumes that countries are more strongly connected if they are more likely to belong to the same cluster within the respective layers. To this aim, we introduce a perturbed distance measure based on the clustering similarity of the countries while penalizing the dissimilarity in at least one of the layers. We employ a fuzzy silhouette method to determine the optimal number of clusters for each year. This helps refine the clustering results and ensures the most meaningful partitioning of countries within each layer.

This methodology provides an interesting framework for understanding the dynamics of technological innovation in EU countries. Indeed, results give a clear view of TI in the EU context and for the analyzed period, emphasizing the deviations and the regularities among individual countries over the considered period. Some conjectures on the link between the main systemic events over the investigated years and the policies on innovations at the EU level can be also advanced, even if they deserve further investigation (see the next section for a discussion on this point). Moreover, the methodology offers a flexible model that can be applied to several real-world data for several contexts.

### 5.1 Limitations and future research

Regarding potential avenues for future research, we might consider an in-depth study on the optimal number of clusters identified through the silhouette function. The main motivation for this exploration can be found in the great heterogeneity of countries in the observed variables in conjunction with significant geopolitical or economic events like the COVID-19 pandemic—which emerged in late 2019 and potentially triggered a global economic recession—followed by the National Recovery and Resilience Plan (NRRP)—which officially began in 2022. It would therefore be of great interest to explore whether there is a causal relationship between these factors. Furthermore, it would be interesting to examine whether the optimal number of clusters influences the intensity of connections between countries across different variables. Finally, a sensitivity analysis of the balance parameter *α* could be exploited in the identification of the optimal level of *α* that guarantees the maximum possible realistic representation of the data.

There are, however, certain limitations to this approach. The most prominent one is the computational complexity of the numerical procedure. Indeed, the complexity of the model would grow significantly as the number of nodes and variables increases, making both the representation of results and their interpretation more challenging. In particular, a larger dataset could lead to computational issues, especially in terms of processing time and memory requirements. Additionally, the clustering results might become less stable as the dimensionality of the data increases, which could affect the reliability of the analysis. Another limitation lies in the scope of the variables considered. While our study focused on a limited set of geopolitical and economic indicators, expanding this to include additional variables—such as cultural or social factors—might provide a more comprehensive view, but would also introduce additional complexities in modeling and interpretation.

## Supporting information

S1 File Data and replication code. (ZIP)
